# Neural correlates of moderate hearing loss: time course of response changes in the primary auditory cortex of awake guinea-pigs

**DOI:** 10.3389/fnsys.2014.00065

**Published:** 2014-04-28

**Authors:** Chloé Huetz, Maud Guedin, Jean-Marc Edeline

**Affiliations:** Centre de Neurosciences Paris-Sud, CNRS, UMR 8195, Université Paris-SudOrsay, France

**Keywords:** acoustic trauma, vocalization, animal, spike timing, tuning curve, single unit recording

## Abstract

Over the last decade, the consequences of acoustic trauma on the functional properties of auditory cortex neurons have received growing attention. Changes in spontaneous and evoked activity, shifts of characteristic frequency (CF), and map reorganizations have extensively been described in anesthetized animals (e.g., Noreña and Eggermont, 2003, 2005). Here, we examined how the functional properties of cortical cells are modified after partial hearing loss in awake guinea pigs. Single unit activity was chronically recorded in awake, restrained, guinea pigs from 3 days before up to 15 days after an acoustic trauma induced by a 5 kHz 110 dB tone delivered for 1 h. Auditory brainstem responses (ABRs) audiograms indicated that these parameters produced a mean ABR threshold shift of 20 dB SPL at, and one octave above, the trauma frequency. When tested with pure tones, cortical cells showed on average a 25 dB increase in threshold at CF the day following the trauma. Over days, this increase progressively stabilized at only 10 dB above control value indicating a progressive recovery of cortical thresholds, probably reflecting a progressive shift from temporary threshold shift (TTS) to permanent threshold shift (PTS). There was an increase in response latency and in response variability the day following the trauma but these parameters returned to control values within 3 days. When tested with conspecific vocalizations, cortical neurons also displayed an increase in response latency and in response duration the day after the acoustic trauma, but there was no effect on the average firing rate elicited by the vocalization. These findings suggest that, in cases of moderate hearing loss, the temporal precision of neuronal responses to natural stimuli is impaired despite the fact the firing rate showed little or no changes.

## Introduction

Over the last decade, an increasing number of studies have described the reorganizations occurring in the adult auditory system after partial hearing loss performed either by traumatic noise or by partial lesions of the cochlea (e.g., Robertson and Irvine, 1989; Kamke et al., 2003; Noreña and Eggermont, 2003, 2005; Rosen et al., 2012). At the thalamocortical level, electrophysiological studies have documented that exposure to loud, traumatic sounds generating partial hearing loss produced alterations in frequency tuning (Rajan, 1998, 2001; Kimura and Eggermont, 1999; Noreña and Eggermont, 2003; Scholl and Wehr, 2008; Gourévitch and Edeline, 2011) and tonotopic map reorganizations (Noreña and Eggermont, 2005). These changes in functional properties may result both from physiological modifications already occurring at subcortical levels (Wang et al., 1996, 2002; Kamke et al., 2003; Vale et al., 2003) and/or from morphological alterations of cortical cells such as modifications in dendritic morphology and in dendritic spine numbers (Fetoni et al., 2013).

In all but three cases (Sun et al., 2008; Noreña et al., 2010; Rosen et al., 2012), the electrophysiological experiments performed so far after hearing loss have assessed the functional properties of auditory cortex neurons under general anesthesia a few hours (Kimura and Eggermont, 1999; Noreña and Eggermont, 2003) to a few days (Rajan, 1998, 2001) or months (Robertson and Irvine, 1989; Gourévitch and Edeline, 2011) after hearing loss. The apparent discrepancy between all these results might be simply due to the use of different delays between the beginning of hearing loss and the time at which the recordings are collected. More precisely, if neuronal activity is collected shortly after hearing loss, it will reveal a neuronal correlate of temporary threshold shift (TTS), whereas recording neural activity months after hearing loss will reveal a correlate of permanent threshold shift (PTS) with a potential contribution of functional reorganizations occurring at the cortical and subcortical levels.

In the present study, we aimed at following the time course of cortical changes from day to day after an acoustic trauma. Single neuron activity was recorded from micro-electrodes chronically implanted in the primary auditory cortex of awake guinea pigs and the responses of cortical neurons were tested from 3 days before to 15 days after the acoustic trauma. On a subset of cells, we also recorded responses to conspecific vocalizations before and up to 3 days after the acoustic trauma.

## Materials and methods

### Subjects and surgery

Experiments were performed on 10 adult (3–6 month old, 5 males, 5 females) pigmented guinea pigs (390–650 g) with a national authorization N°91–271 to conduct animal research and protocol specifically approved by the CNRS and Paris-Sud University (CEEA, Ethic committee N°59). The animals were housed in a colony room and were grouped by four or five animals in large plastic cages (75 × 55 × 25 cm; Tecniplast, Buguggiate, Italy) with large wire mesh doors (55 × 20 cm). All animals frequently emitted vocalizations during social interactions with the other animals of the same cage and loudly vocalized during animal care and feeding. We did not notice obvious changes in behavior or in amount of emitted vocalizations before vs. after the acoustic trauma.

Initially, the animals underwent a sterile surgery under anesthesia (atropine 0.08 mg/kg, diazepam 8 mg/kg, pentobarbital 20 mg/kg; see Evans, 1979). Three silverball electrodes were inserted between the bone and dura: one was used as a reference during the recording sessions; the other two, placed over the frontal and parietal cortices, served to monitor the cortical electroencephalogram (EEG) during the subsequent recording sessions. A large opening was made in the temporal bone and very small slits (200 μm) were made in the dura matter under microscopic control. A diagram of the vasculature pattern was drawn and the primary field (AI) location was estimated based on those observed in our previous studies (Edeline and Weinberger, 1993; Manunta and Edeline, 1999; Edeline et al., 2001; Huetz et al., 2009). A coarse mapping of the cortical surface was made to confirm the location of AI: neuronal clusters were recorded with low impedance (<1 MW) electrodes until a progression from low to high frequency was observed in the caudo-rostral direction (Wallace et al., 2000; Gaucher et al., 2012). An array of 5 tungsten electrodes (~1.0 MΩ at 1 kHz, spaced 200–300 μm in the rostro-caudal axis) was slowly inserted in the auditory cortex under electrophysiological control. Starting from 600 μm below the pia, responses to pure tone frequencies were tested at regular depths to optimize the strength of evoked responses; the final placement depth of the electrodes ranged from 800 to 1250 μm which correspond to cortical layers III/IV (Wallace and Palmer, 2008).

A dental acrylic cement pedestal, including two cylindrical threaded tubes, was built to allow for atraumatic fixation of the animal's head during the subsequent recording sessions. An antiseptic ointment (Cidermex, neomycine sulfate, Rhone-Poulenc Rorer) was liberally applied to the wound around the pedestal. An injection of non-steroidal anti-inflammatory (Tolfedine 0.1 mg/kg) was given at the end of the surgery and the 2 following days. All surgical procedures were performed in compliance with the guidelines determined by the National (JO 887-848) and European (86/609/EEC) legislations on animal experimentation, which are similar to those described in the *Guidelines for the Use of Animals in Neuroscience Research of the Society of Neuroscience.* Regular inspections of our laboratory by accredited veterinarians designated by the CNRS and Paris-Sud University confirmed that care was taken to maximize the animals' health and comfort throughout the different phases of the experiment.

### Recording procedures

Three days after surgery, each animal was adapted to restrained conditions in an acoustically isolated chamber (IAC, model AC2) for several days. The animal was placed in a hammock with the head fixed for increasing periods of time (from 10–20 min to 1–2 h per day). The animal was also accustomed to hearing sequences of pure tone bursts as well as different vocalizations used subsequently to test the neuronal responses. At least 4 days of adaptation of restrained conditions were allowed before the collection of neuronal recordings.

The recording procedures were the same as in previous studies (Edeline et al., 2000, 2001; Huetz et al., 2009). The signal from the electrode was amplified (gain 10000; bandpass 0.6–10 kHz,) then multiplexed in an audio monitor and a voltage window discriminator. The action potentials waveform and the corresponding TTL pulses generated by the discriminator were digitized (50 kHz sampling rate, Superscope, GW Instruments), visualized on-line and stored for off-line analyses. The pulses were sent to the acquisition board (PClab, PCL 720) of a laboratory microcomputer, which registered them with a 50 μs resolution and provided on-line displays of the neuronal responses. For each animal, the signal from each electrode was tested daily and the data collection only started when, under 1–3 electrodes, action potential waveforms can be unambiguously attributed to a single neuron.

### Audiometry and exposure to the traumatic sound

Auditory brainstem responses (ABRs) were recorded as previously described (Gourévitch et al., 2009; Gourévitch and Edeline, 2011). Briefly, ABR were recorded via subcutaneous electrodes (SC25, Neuro-Services); using a Centor-USB interface and software (DeltaMed, France). The signal was filtered (0.2–3.2 kHz, sampling rate 100 kHz), waveforms were averaged (500–1000 waveforms depending on the stimulus intensity) and stored for off-line analyses on a computer. An artifact rejection procedure was used during averaging, the rejection criterion being ±40 μV. The stored waveforms were examined and the threshold was defined as the lowest level (dB re:20 μPa) at which a clear waveform could be observed. Intensity levels were always below 90 dB in order to avoid inducing additional hearing loss on traumatized animals.

Hearing loss was realized by a single 1 h exposure to a loud sound. The animals, placed individually in a wire mesh cage (23 × 23 × 15 cm), were exposed to a traumatic tone (pure tone of 5 kHz at 110 dB SPL) in an acoustically isolated chamber (IAC model AC2). The pure tone was generated by the wave generator (Hewlett-Packard, model HP 8903B), amplified (Prism-Audio, model LA-150M) and sent to two piezoelectric tweeters (Motorola, model KSN 1005) located on each side of the cage. The sound delivery system was calibrated to obtain 110 ± 10 dB at various locations in the cage using a calibrated type I precision sound level meter (B&K model 2235). A videocamera, installed in the acoustic chamber, allowed visualizing the animal during exposure and checking that there was no preferred orientation of the animal regarding the two speakers. During the 5 first minutes of exposure, freezing behavior was observed most of the animals (*n* = 8), then the animals moved toward a corner of the cage and stayed there for the rest of the exposure. In two other cases, the animal explored the cage and moved during the 5 first minutes then stayed at the same location in the cage. During the rest of the 1 h exposure, we did not observed particular signs of stress, panic, or abnormal behavior. The amount of excretion (urine and feces) found in the cage at the end of the 1 h exposure session was not different from what is usually found when guinea pigs are placed for 2 h in a new environment (Manunta and Edeline, 1999; Tith and Edeline, unpublished data).

### Auditory stimuli and experimental protocol

All the cells included in the present study exhibited reliable tuning curves when tested with pure tone frequencies. The sound generating system to deliver pure tone frequencies was the same as that previously described, (Edeline et al., 2001; Manunta and Edeline, 2004; Huetz et al., 2009). Pure tones (100 ms, rise/fall time 5 ms) were generated by a remotely controlled wave analyzer (Hewlett-Packard model HP 8903B) and attenuated by a passive programmable attenuator (Wavetek, P557, maximal attenuation 127 dB), both controlled via an IEEE bus. Stimuli were delivered through a calibrated earphone (Beyer DT48) placed close to the ear canal. The system was calibrated using a sound level calibrator and a condenser microphone/preamplifier (Bruel and Kjaer models 4133 and 2639T) placed at the same distance from the speaker as the animal's ear (<5 mm). The whole sound delivery system (HP 8903B, attenuators, and speaker) was calibrated from 0.1 to 35 kHz and could deliver tones of 80 dB up to 20 kHz and of 70 dB up to 35 kHz. Harmonic distortion products were measured to be down about 50 dB from the fundamental. The EEG (bandpass 1–90 Hz) was displayed on a polygraph (Grass, model 79D) to make sure that the animal was awake during the entire recording session (the data collection was stopped when large voltage EEG signals characteristic of slow-wave sleep were present).

When the recordings were stable enough and the animal quiet enough after completion of the tuning curve determination, three typical guinea pig vocalizations (Berryman, 1976; Harper, 1976) used in our previous studies (Philibert et al., 2005; Huetz et al., 2009) were presented in their natural and time-reversed versions. These vocalizations were collected from five adult male guinea pigs recorded either in pairs or individually in a sound attenuated room. Calls were recorded using a Sennheiser MD46 microphone connected to a microcomputer and digitized using SoundEdit software (44 kHz sampling rate). The relationships between these calls and the animal behavioral repertoire have been previously described (Berryman, 1976; Harper, 1976). A “chirp” is a brief call (0.7–15 kHz, <100 ms) that is believed to be a low-intensity distress call or a warning signal. A “chutter” consists of a chain of five components (0.5–3.5 kHz, 150–250 ms separated from each other by 140–175 ms) emitted during discomfort. A “whistle” is a two-part call (250–400 ms, with the first part from 1–3 kHz and the second part rising steeply to 8–20 kHz) emitted when animals are isolated or in response to stimuli associated with caretaking. The time-reversed versions of the stimuli were generated by reversing the natural calls in the time domain, i.e., playing the call backward. Each call was presented at a peak intensity of 70 dB sound pressure level. The synchronization between the vocalizations' onset and the spike trains was made by a synchronization pulse triggered when the vocalization intensity crossed a voltage threshold. Therefore, the neuronal recordings to different vocalizations were not synchronized with the real onset of each vocalization but rather with a fixed sound pressure level reached by the vocalization. The natural and time-reversed versions of the three calls were presented in random order, with each call repeated 20 times with a 2-s period of silence between each vocalization. The whole protocol, i.e., testing the frequency tuning with pure tones and the responses to the four vocalizations (natural and time-reversed) lasted approximately 60 min. The EEG was displayed on a polygraph and a computer to make sure that the animal was awake during the entire recording session. The animals did not vocalize during the recording sessions. Neuronal activity was recorded during recording sessions separated by 24 h from 3 days before up to 15 days after the trauma. In all cases, the recording session was stopped each time the spike waveform became unstable. Systematic off-line examination of the digitized waveforms confirmed that spike trains of unambiguously isolated single units were recorded.

### Histological analyses

After the last recording session, the animals received a lethal dose of pentobarbital (200 mg/kg), and small electrolytic lesions were made by passing anodal current (10 μA, 10 s) through the recording electrodes. The animals were perfused transcardially with 0.9% saline (200 ml) followed by 2000 ml of fixative (4% paraformaldehyde in 0.1 M phosphate buffer, pH 7.4). The brains were subsequently placed in a 30% sucrose solution for 3–4 days; then coronal sections were cut on a freezing microtome (50 μm thickness) and counterstained with cresyl violet. The analysis of histological material was always done blind of the electrophysiological results. The sections were examined under several microscopic magnifications to find the electrode tracks corresponding to the implanted tungsten electrodes. Determinations of the relative thickness of cortical layers in the guinea-pig Auditory Cortex (ACx) (Wallace and Palmer, 2008) were used to assign each recording to a cortical layer.

### Data analysis

For each cell, the frequency tuning was quantified from threshold up to 80 or 70 dB by 10 dB steps. At each intensity, the best frequency was determined as the frequency eliciting the largest evoked responses. The breadth of tuning was quantified by the Q_20 dB_ (but the Q_10 dB_ and the Q_40 dB_ were computed as well). The latency of the tone-evoked responses was computed at each intensity used to test the frequency tuning curve. At a given intensity, the responses obtained for all the tested frequencies were considered, and the latency of the first spike after tone onset was computed (1-ms precision). For each cell, at each intensity, the variability of the latency was quantified by the standard deviation of the mean latency value.

For each cell, the responses to the vocalizations were analyzed in terms of evoked firing rate and spike timing reliability. Since the three tested vocalizations had different lengths (from 90 ms for the “chirp,” up to 1740 ms for the “chutter”), only the first 90 ms were analyzed to allow pooling of the responses to different vocalizations. The spike timing reliability was computed using the *CorrCoef* as in previous studies (Gaucher et al., 2013a). It corresponds to the normalized covariance between each pair of spike trains recorded at presentation of this vocalization and was calculated as follows:

$$CorrCoef=\frac{1}{N(N-1)}\sum_{i=1}^{N-1}\sum_{j=i+1}^{N}\frac{\sigma x_{i}x_{j}}{\sigma x_{i}\sigma x_{j}}$$

where *N* is the number of trials and σ*x*_*i*_*x*_*j*_ is the normalized covariance at zero lag between spike trains *x*_*i*_ and *x*_*j*_ where *i* and *j* are the trial numbers. Spike trains *x*_*i*_ and *x*_*j*_ were previously convolved with a 10 ms half-width Gaussian window.

Onset PSTHs were constructed by summing up all the trials of all cells recorded on each day before and after the hearing loss. These PSTHs were constructed only for the onset response, i.e., for the first 90 ms after the beginning of each vocalization. They were made for the days on which a sufficient number of recordings was available (from day −3 to day +3). They were first computed for each vocalization, but as the main result was similar for all vocalizations, the figures show averaged onset PSTH over all vocalizations.

In the following text and figures, days before and after trauma are labeled D−X or D+X (X being the number of days before −, or after + the acoustic trauma).

## Results

### Evaluation of the hearing deficit by ABR

**Figure 1:**
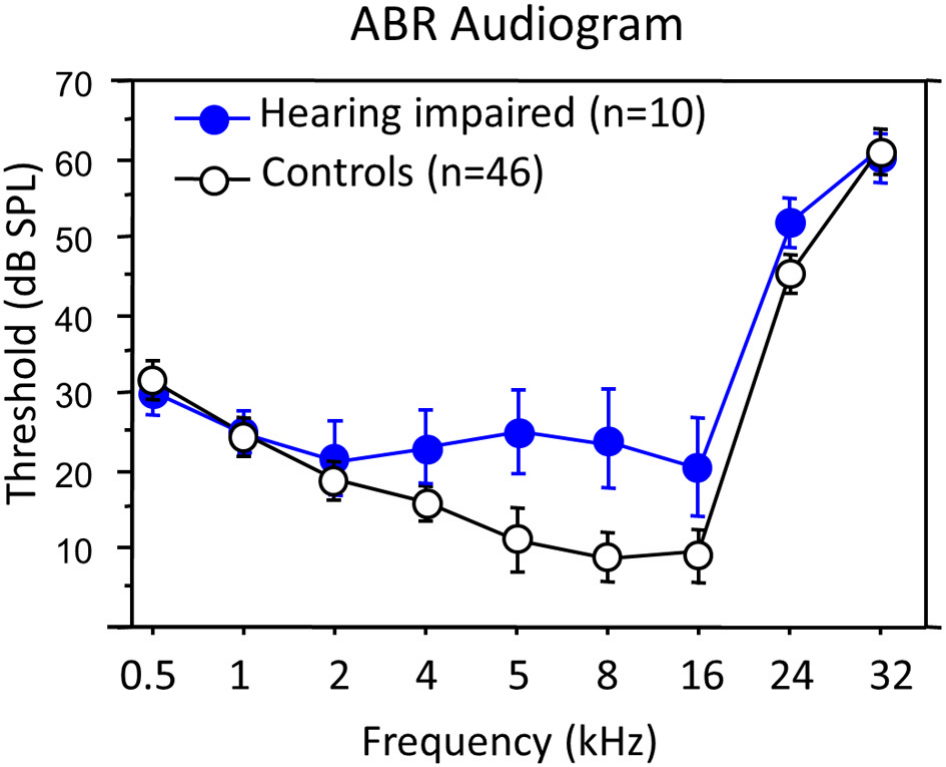
**Average audiogram of hearing impaired animals as compared to control animals**. Blue dots represent the averaged threshold of the ABR audiogram of the animals (*n* = 10) included in this study, and the error bars indicate the standard error of the mean. White dots are computed from a database of ABRs obtained in control guinea pigs (*n* = 46) of similar age and weight.

Since we did not record the ABRs of our animals (*n* = 10) before and after the acoustic trauma, hearing loss evaluation is only based on ABRs obtained a few weeks (between 4 and 8 weeks) after the last recording session. We compared the ABRs obtained from our hearing impaired animals with a large database of ABRs obtained (with exactly the same equipment) in control guinea pigs (*n* = 46) of the same age (4.7 ± 0.6 months) and weight (585 ± 113 g) than the ones used here. The control animals had typical ABR audiograms similar to those previously published (Gourévitch et al., 2009; Gourévitch and Edeline, 2011). Compared to control animals, the animals used in the present experiment had a consistent hearing deficit (15–20 dB on average) in the 4–8 kHz range (Figure 1). Statistical analyses confirmed that there was no hearing deficit in the low frequency range (unpaired *t*-tests, *p* > 0.10 at 0.5, 1 and 2 kHz), a clear and significant hearing deficit in the mid frequency range (*p* < 0.01 for 4, 5, and 8 kHz). The hearing loss was modest but still significant at 16 kHz (*p* = 0.047); it was not for 24 and 32 kHz (*p* > 0.08 in both cases). Thus, the long-term effects of the exposure were mostly an increased threshold of 15 dB at, and one octave above, the trauma frequency. Most likely, these changes should be considered as a PTS since they were obtained 1.5–2.5 months after the acoustic trauma.

### Consequences of the tuning curves parameters

Thirty-two cortical sites were studied in eight animals from 3 days before to 15 days after the acoustic trauma (for two animals none of the electrodes gave satisfactory signal-to-noise ratio to record clear single unit activity over the 2 weeks of recording). On each recording session, special cares were taken to make sure that the discharges of a single unit were actually recorded. From 1 day to the next, it was not possible to determine whether or not the same neuron was recorded and, rather than claiming that the same cells were recorded over time, we prefer to consider that it was the same cortical site from which cells were sampled across the 3 weeks of the protocol.

**Figure 2:**
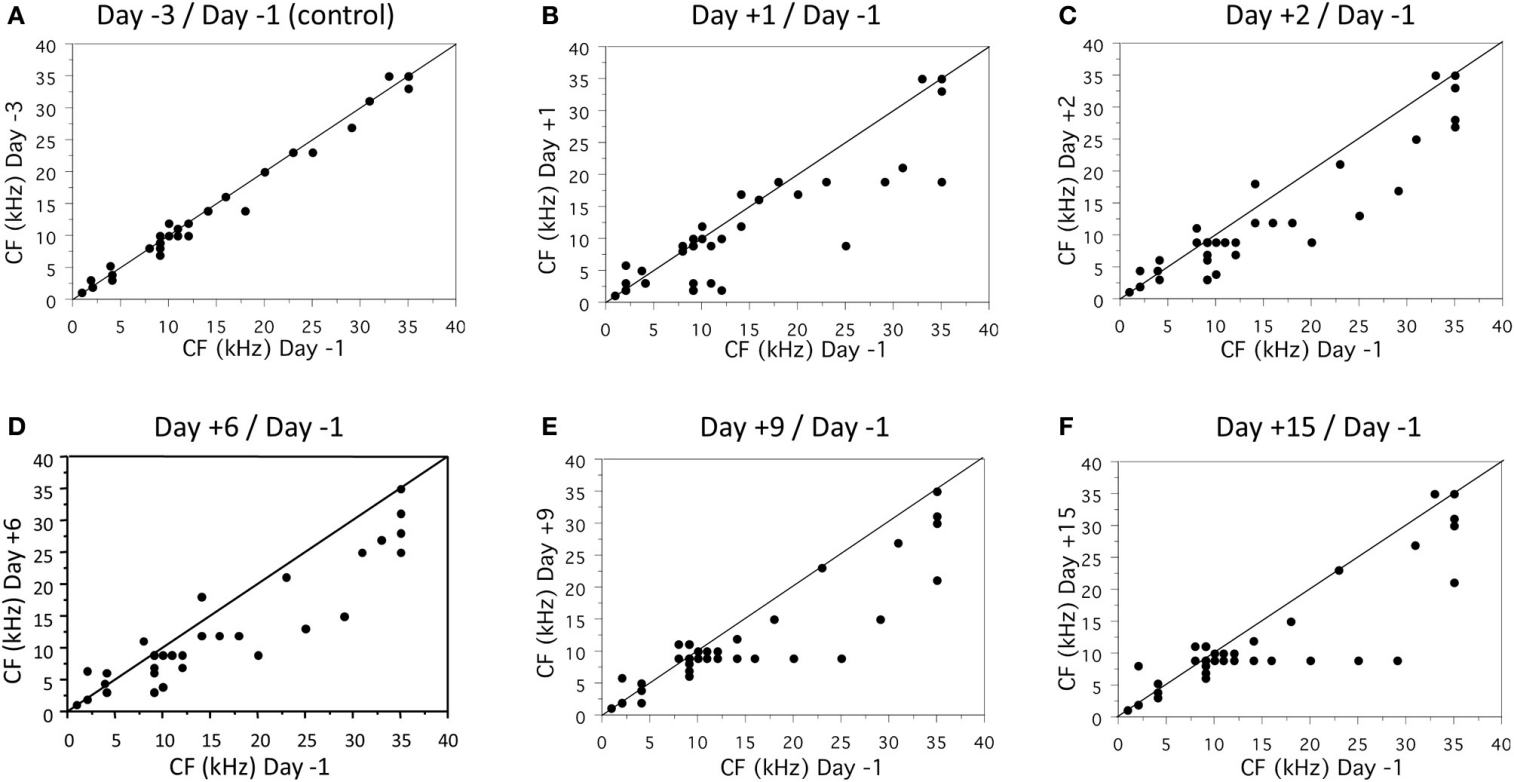
**Time course of Characteristic Frequency shifts from 1 day to 15 days after the trauma**. Each scatterplots represent the Characteristic Frequency (CF) of the recorded neurons on the day of interest (y-axis) against a control value computed the day before the trauma (x-axis). The day of interest (y-axis) corresponds to 3 days before trauma **(A)**, and 1 day **(B)**, 2 days **(C)**, 6 days **(D)**, 9 days **(E)**, and 15 days **(F)** after trauma. Dots below the diagonal black lines correspond to neurons for which the CF was decreased after the trauma.

**Figure 3:**
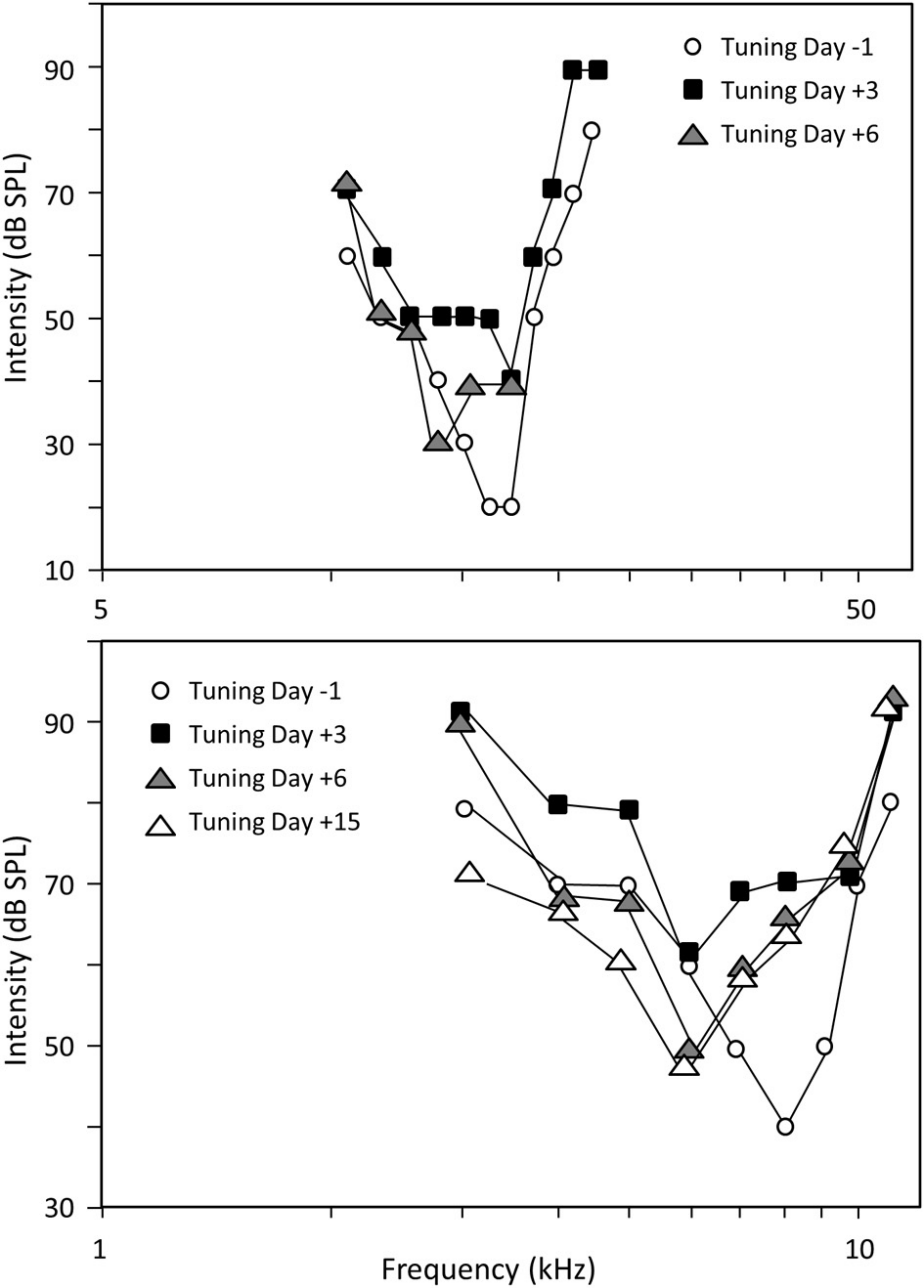
**Frequency shifts of two cortical tuning curves**. Frequency tuning curves of two individual neurons (top and bottom). For each neuron, the tuning curve is represented the day before the trauma (white circles), 3 days (black square) and 6 days (gray triangle) after the trauma. The decrease in threshold value at CF from D+3 to D+6 might reflect a putative transition between TTS and PTS.

**Figure 4:**
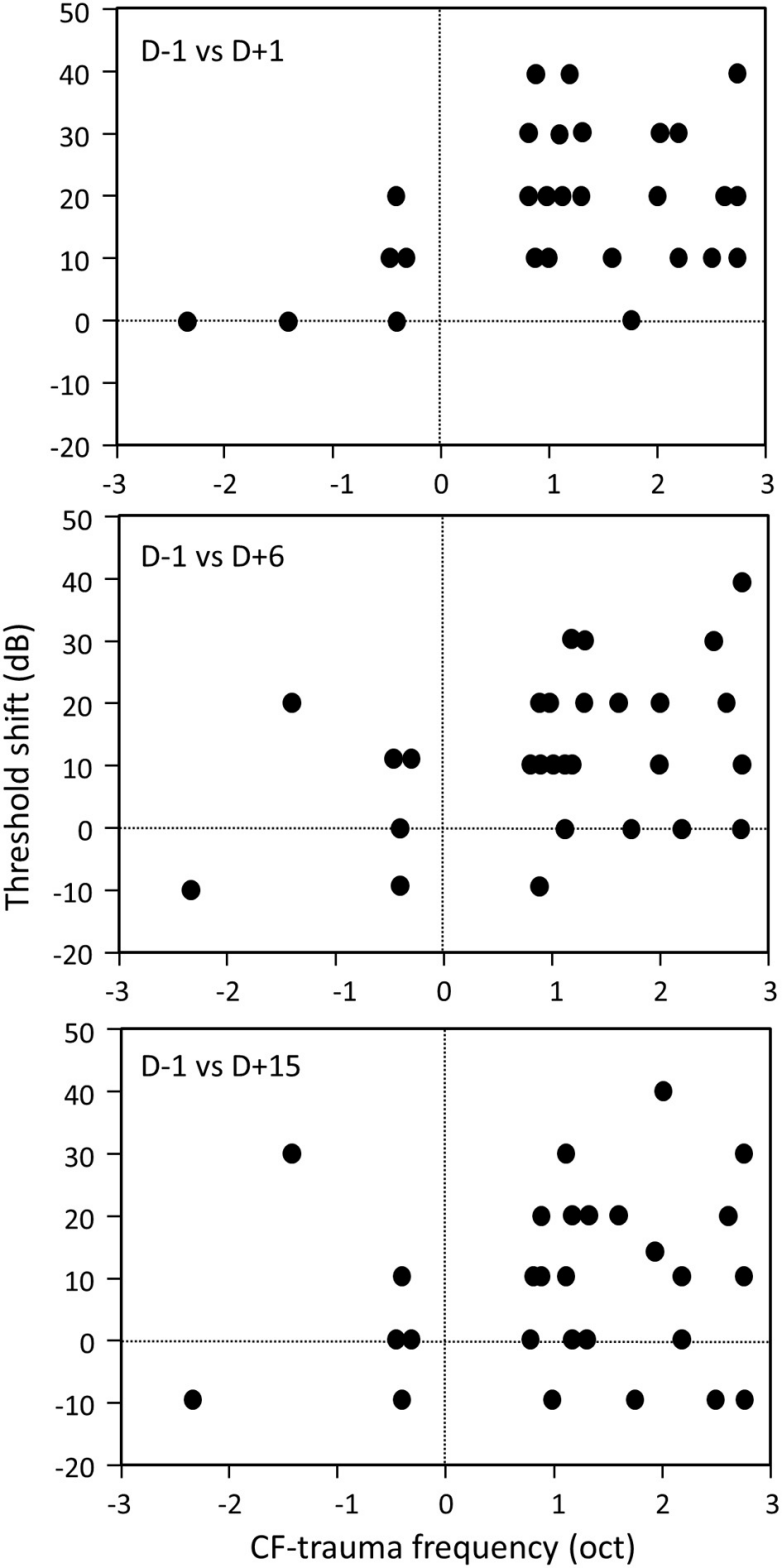
**Lack of correlation between ABR threshold shifts and CF distance from trauma frequency**. Each scatterplot represents the ABR threshold shift as a function of the frequency shift (e.g., CF—trauma frequency) of each neuron 1 day (top), 3 days (middle), and 6 days (bottom) after the trauma.

On a given recording session, the neuronal responses were determined at 3–7 intensities (from 80 or 70 dB to threshold) thus allowing quantifications of functional parameters. The scattergrams presented on Figure 2 display the characteristic frequency (CF) derived at each cortical site before trauma vs. after trauma. Comparing the CF obtained 3 days vs. 1 day before the acoustic trauma (Figure 2A) indicates that there was a relative good match between the CF values, which suggests that there was a decent amount of stability of the CF in control conditions. During all the following recording sessions after the trauma, the general tendency was the same: cortical sites with initial CF above 8 kHz displayed lower CF after the trauma (dots below the diagonal lines in Figures 2B–F). Statistical analyses indicated that there was no change in CF value between two recording sessions before the acoustic trauma (*p* > 0.32), whereas there was a significant decrease of the CF values from the first (D+1) to the last (D+15) recording session after the trauma (paired *t*-test, *p* < 0.05 in all cases). This effect is illustrated on Figure 3: For two different cortical sites, the tuning curves clearly display a shift of at least one octave in the low frequency range. Note that there was a partial recovery of the threshold between the first day and the third day after the trauma. Also, as shown in Figure 4, there was no correlation between the threshold shifts and the shifts in CF values: even at D+15, cells within the same frequency band can display either large increase in threshold or a small decrease in threshold.

**Figure 5:**
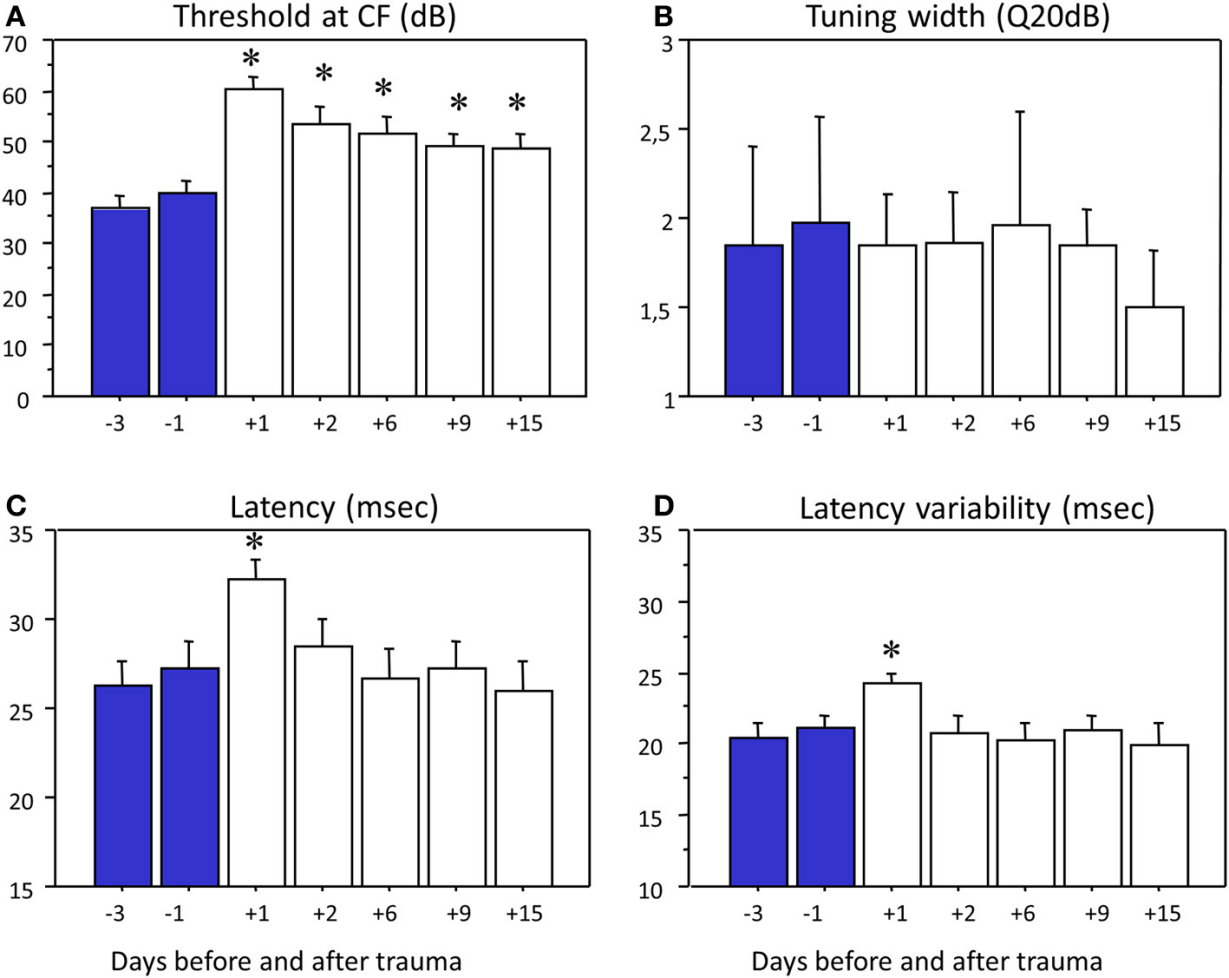
**Impact of hearing loss on the tuning curve parameters**. The histograms display the averaged values of the threshold at CF **(A)**, tuning width **(B)**, latency **(C)**, and latency variability **(D)** across days before and after trauma (from 3 days before to 15 days after the trauma). Values before trauma are indicated in blue, whereas values after trauma are in white. The error bars show the standard error of the mean. The decrease in threshold value at CF from D+1 to D+9 might reflect a dissipation of the TTS effect and the stabilization of threshold value at CF between D+9 and D+15 could suggest that a PTS is reached. Stars correspond to statistically significant difference (*p* < 0.05) from control level at Day-1.

Based on the tuning curves obtained at each cortical site before and after the trauma, the group data clearly pointed out that there was an increase in cortical threshold. Figure 5A shows the evolution of the mean threshold value from 3 days up to 15 days after the acoustic trauma. There was a 20 dB increase (from 39.6 to 61.4 dB, *p* < 0.01) in threshold when comparing the day preceding the trauma and the day following the trauma. In the following days, this increase in threshold was less pronounced (10 dB on average) but the mean threshold remained significantly higher than before trauma (*p* < 0.05 at 9 and 15 days post-trauma). It tended to stabilize after the trauma since there was not threshold difference at 9 and 15 days post-trauma (*p* > 0.20).

As shown in Figure 5B, the quantification of the tuning width by the Q_20 dB_ revealed neither systematic tuning curve enlargement nor systematic shrinkage after the acoustic trauma (all *p*-values > 0.18). Quantification of the Q_10 dB_ or the Q_40 dB_ did not reveal effect that could have been masked by the arbitrary choice of quantifying the tuning curve at a particular intensity (*p* > 0.15 in all cases). Analyzing the tuning width according to the CF frequency did not reveal more effects: There were no statistical differences in tuning width for cells with low CF (<5 kHz), middle CF (5 < CF < 10 kHz) and high frequency CF (>10 kHz).

In contrast, quantification of the latency of the tone evoked responses revealed a marked effect (Figure 5C): At the highest intensity tested (70 or 80 dB), there was a large and significant (*p* < 0.01) increase in response latency the first day following the acoustic trauma (from 27.2 to 32.5 ms). This increase in latency was no longer present the subsequent days (*p* > 0.25 in all cases). Similarly, the variability of the response latency was increased the first day after the acoustic trauma (*p* < 0.05, Figure 5D) but not the following days (*p* > 0.27 in all cases). In fact, subsequent analyses revealed that this increase in response latency and latency variability was present for the cells exhibiting a CF above 5 kHz before the trauma (*p* < 0.01) but was less pronounced for the cells exhibiting lower CF (*p* = 0.07). Note that this increase in latency and latency variability was still significant (*p* < 0.05) when the responses obtained at all the tested intensities (80–20 dB) were pooled together.

To summarize, the quantification of the tuning curves indicated that there was a 20 dB increase in cortical threshold in the 24 h following the acoustic trauma accompanied by a 5 ms mean increase in response latency and in variability of response latency. Although attenuated, the increase in cortical auditory threshold was still present up to 15 days post-trauma, but the changes in latency and latency variability could no longer be detected after the first day post-trauma. Whatever the frequency band that was considered (below the trauma frequency, within 1.5 octave above, or more than 1.5 octave above it), there was no correlation between the ABR threshold shift and the cortical threshold shift (lowest *p*-value, *p* = 0.23). This lack of relationship was previously reported (see Figure 4 in Gourévitch and Edeline, 2011).

### Alterations of the responses to conspecific vocalizations

**Figure 6:**
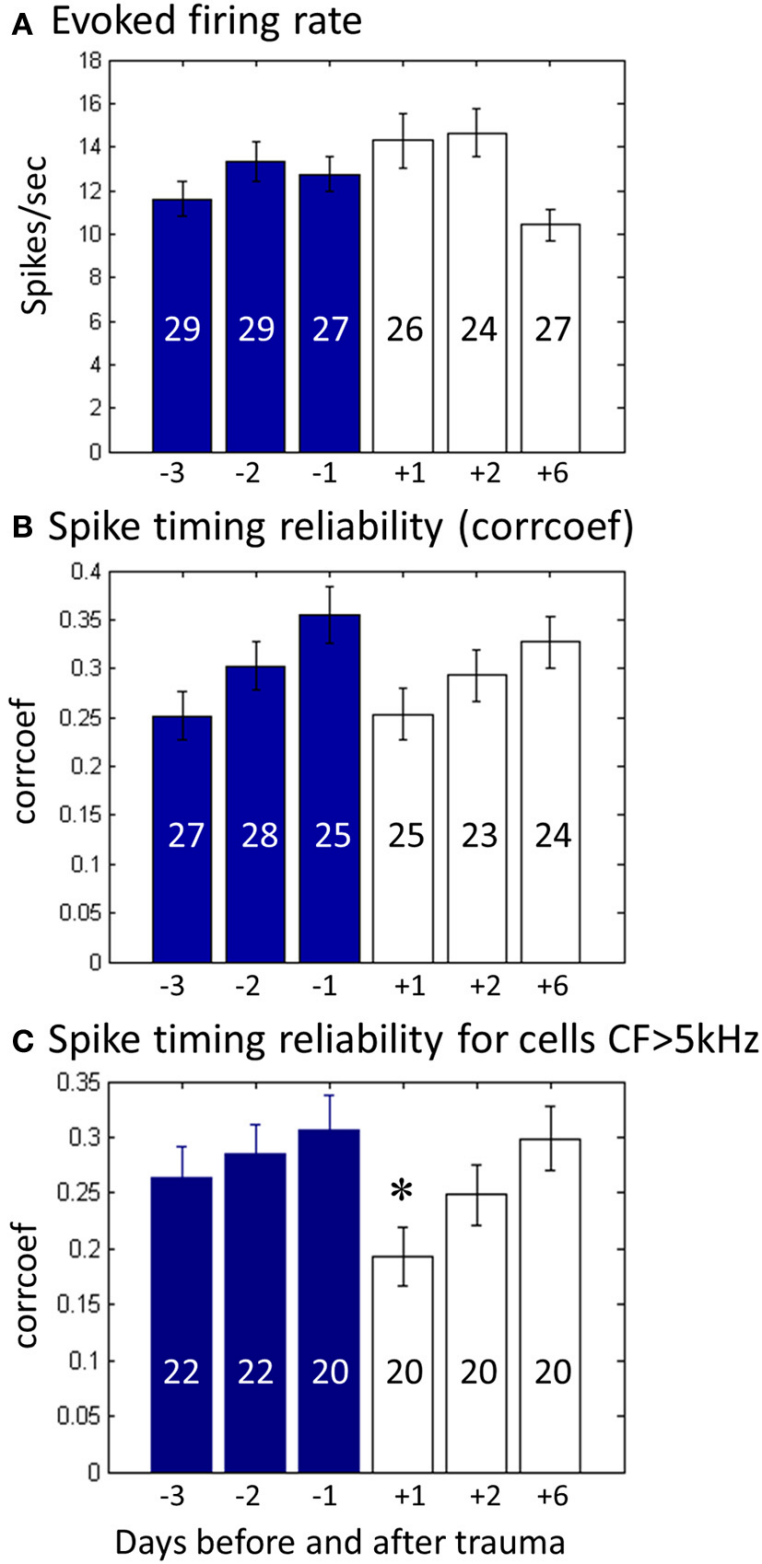
**Impact of hearing loss on the responses to vocalizations. (A)** Histogram of the average firing rate during the first 90 ms of the responses to the different vocalizations from 3 days before trauma (blue) to 3 days after (white). The number of neurons in each recording day is indicated within each bar. **(B)** Same for the spike timing reliability as computed from the *CorrCoef* (see Methods) for all recorded neurons. The *CorrCoef* is an index of the trial-to-trial reliability of the response. **(C)** Same as **(B)**, but only for neurons which CF is above the trauma frequency (CF > 5 kHz). Stars correspond to statistically significant difference (*p* < 0.05) from control level at Day-1.

Each time the animal was still quiet after the completion of the tuning curve determination, a set of previously used conspecific vocalizations (Philibert et al., 2005; Huetz et al., 2009) was presented at 70 dB SPL (peak amplitude) in their normal and time-reversed versions. Based upon previous studies (review in Huetz et al., 2011; Gaucher et al., 2013b), two parameters were quantified to assess the effect of hearing loss on the responses to communication sounds, the firing rate and the spike timing reliability of the responses. On average, we could not detect significant change in evoked firing rate during the presentation of the vocalizations at 70 dB. Figure 6A displays the evoked firing rate for the 3 days before and the 3 days after the acoustic trauma: There was a slight increase in evoked response the 2 first days after the trauma, and a non-significant decrease on the third day. None of these variations were significant as they were in the range of the pre-trauma response fluctuations. Similarly, we could not detect significant effect on the spontaneous firing rate. Even if an ANOVA across days was significant (*F* = 7.14, *p* < 0.01), the difference between D−1 (3.1 spikes/s) and D+1 (3.9 spikes/s) was smaller than the difference between D-2 (4.7 spikes/s) and D−1. Thus, day-to-day fluctuations in spontaneous firing rate before the acoustic trauma might have prevented to detect significant effects after the acoustic trauma.

The *CorrCoef* index, i.e., the index quantifying the spike timing reliability of evoked responses, did not indicate significant changes (Figure 6B). There was a small decrease in spike timing reliability on the 2 first days after the acoustic trauma, but, as for the firing rate, this change was in the range of the pre-trauma fluctuations (ANOVA, *F* = 1.82, *p* = 0.10). For the cells with CF above 5 kHz, we did not detect significant changes in terms of evoked firing rate. However, for these cells the *CorrCoef* index showed a significant decrease on the first day after the trauma compared to the day before trauma (ANOVA, *F* = 2.34, *p* = 0.04, paired *t*-test between D−1 and D+1, *p* = 0.007) suggesting that the acoustic trauma transiently impacts the spike timing reliability of middle and high CF cells (Figure 6C).

**Figure 7:**
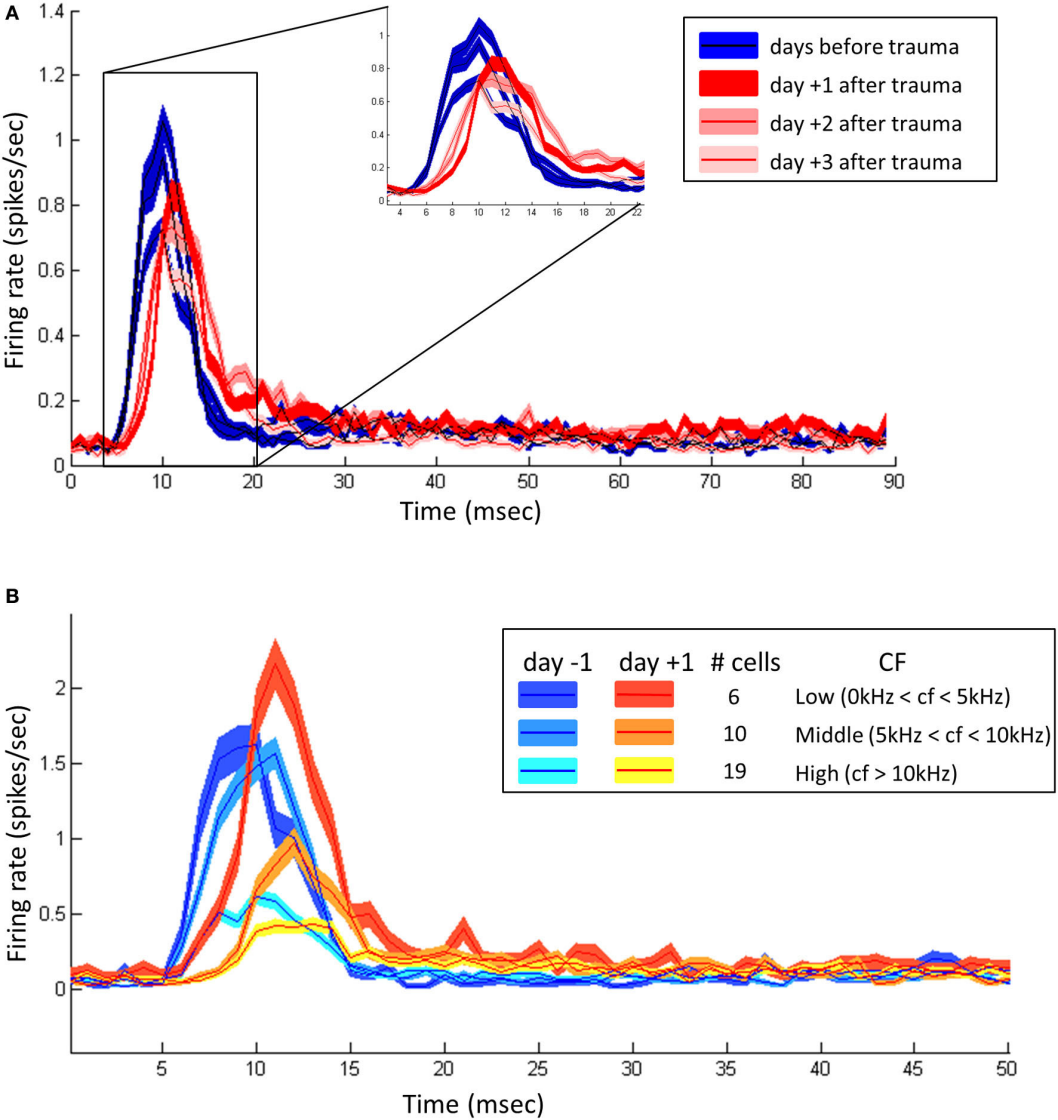
**Impact of hearing loss on the latency and duration of the responses to vocalizations. (A)** Onset Post Stimulus Time Histograms (PSTHs) computed from the responses to the three vocalizations and their time-reversed versions. The blue curves represent the onset PSTHs recorded the 3 days before the trauma (control). The reds curve represent the onset PTSH computed 1 day (deep red), 2 days (light red), and 3 days (lightest red) after the trauma. **(B)** Onset PSTHs computed for three groups of cells (low, middle and high CF) 1 day before and 1 day after the trauma. The inset indicates the color corresponding to each group.

Interestingly, analyzing the average latency obtained from the onset responses to the different vocalizations indicated clear effects. Figure 7A shows average onset PSTHs obtained when pooling the response to the different vocalizations across all the recorded cells. Over the 3 first days before the trauma (blue curves), the latency was relatively stable with a mean latency of 10 ms^1^. During the 3 days after the acoustic trauma (red curves), the latency was increased to a value of 11 ms: Although small in absolute value, this increase in latency was systematic as attested by the shifts of the onset PSTHs. This increase was more pronounced the first day following the trauma then the response latency progressively moved back to control values. The overall shape of the response was also modified. The onset response was reduced and the response duration was increased, suggesting a lack of synchronization of the response at the onset of the vocalizations. Moreover, analyzing the duration of the onset response (by taking a threshold at 0.15 spikes/s which correspond to the baseline level plus 2 standard deviations) revealed that the trauma strongly increased the variability and/or the duration of the onset response: before the trauma, the responses returned to the background firing rate (0.15 spikes/s) after 9 ms, whereas the first day after the trauma, the onset response lasted for 19 ms.

To investigate how the acoustic trauma affects the responses of cells with different CFs, we split the population into three groups according to the cell's CF: the “low CF” group (CF < 5 kHz, *n* = 6) for which the CF was below the trauma frequency, the middle frequency CF (5 kHz < CF < 10 kHz) for which the CF was in one octave above the trauma frequency and high CF (CF > 10 kHz) for which the CF was more than one octave above the trauma frequency. Figure 7B shows the onset PSTHs computed for these three groups the day before and the day after the trauma. Before the trauma, we observed that the higher the CF, the longer the latency and the smaller the onset response. This effect probably results from the fact that there was little energy in the vocalization in the high frequencies, so the strength of the inputs was probably very weak for the neurons with high CF. This pattern was conserved the day following the trauma, but in the three groups, the response latencies was increased after the acoustic trauma (compare the different blue curves with the red-yellow curves). More importantly, for the low CF neurons, only the latency was shifted without any effect on the strength of the onset response. For the two other groups, the trauma not only induced a shift in latency but also a slight decrease of the strength of the onset response.

## Discussion

We show here that an acoustic trauma, producing moderate but permanent hearing loss triggers both transient (D+1) and long-lasting (D+15) events in the responses of auditory cortex neurons. When tested with pure tones, auditory cortex neurons displayed 24 h after the trauma an increase in threshold, accompanied by an increase in latency and latency variability. Only a modest increase in threshold persisted a few days after. When tested with conspecific vocalizations, the responses of cortical neurons displayed a decrease in spike-timing reliability especially for the cells with CF above the trauma frequency, and there was a significant shift in response latency and response duration. These effects clearly suggest that moderate hearing loss impacts on several temporal parameters of neuronal responses in the auditory cortex of awake animals. The immediate effects differ from those observed 15 days later probably reflecting the difference between TTS and PTS.

### Methodological considerations

An obvious pitfall of our experiment is that the ABRs were only recorded at the end of the experiment. Therefore, the hearing loss was assessed by comparing the post-trauma ABRs with a large database of ABR obtained from control animals of the same age and weight. Another consequence was that we could not follow the time course of the ABRs changes in parallel with the changes in cortical responses. However, testing the ABR daily would have required to anesthetize the animals daily (or every other days), which was a risk we did not want to take given that (i) the animals were implanted with chronic micro-electrodes and (ii) they were already adapted to restrained conditions. Despite these limitations, there is clear evidence for hearing loss within one octave at, and above, the trauma frequency. This loss is weaker than in previous studies (Gourévitch et al., 2009; Gourévitch and Edeline, 2011) because of the shorter time of exposure. The increase in cortical thresholds observed here is modest, but it should be kept in mind that even with much larger ABR threshold shifts (about 40 dB), cortical threshold shifts were found to be 5–20 dB (Noreña and Eggermont, 2003; Noreña et al., 2003).

Other limitations are (i) that the size of our population is quite small compared with previous studies performed in anesthetized conditions, and (ii) that we cannot claim that the same neurons were tested before and after trauma. Nonetheless, we sampled the same cortical sites before and after trauma, as it was the case when multiunit recordings were compared before and after trauma (Eggermont and Komiya, 2000; Noreña and Eggermont, 2003; Noreña et al., 2003), which led us to consider that comparison with previous studies is possible.

### Comparison with other studies testing auditory cortex receptive fields after hearing loss

Over the last two decades, several studies have provided compelling evidence for cortical reorganizations after hearing loss (review in Pienkowski and Eggermont, 2011). The origins of hearing loss can be quite diverse ranging from a physical lesion of the cochlea (Robertson and Irvine, 1989; Rajan et al., 1993; Rajan and Irvine, 1996), to intense noise exposure (Eggermont and Komiya, 2000; Noreña and Eggermont, 2003, 2005; Tomita et al., 2004), ototoxic drugs (Harrison et al., 1991; Schwaber et al., 1993), or to genetic pathology (Willott et al., 1993). When the consequences of hearing loss were assessed weeks/months after injury or acoustic trauma, large-scale reorganizations of cortical maps were reported (Robertson and Irvine, 1989; Rajan et al., 1993; Rajan and Irvine, 1996; Eggermont and Komiya, 2000; Noreña and Eggermont, 2005). In contrast, when immediate changes were evaluated, the authors mainly focused on alterations of frequency tuning curves, spontaneous and evoked firing rates (Noreña and Eggermont, 2003; Noreña et al., 2003). Results obtained here in awake animals within a few days after acoustic trauma share some similarities with some of the immediate effects following a trauma. For example, the shifts of CF toward lower values for cells having their original CF above the trauma frequency is similar to what was reported in the cat auditory cortex after a trauma at the same frequency as here (Noreña and Eggermont, 2003; Noreña et al., 2003). However, several other changes described in previous studies were not observed in the present data. For example, we could not detected systematic changes in evoked (Figure 6) or in spontaneous activity after acoustic trauma. In fact, as explained in the Results section, day-to-day fluctuations in evoked and spontaneous activity (either due to differences in the cell types recorded from 1 day to the next by a given electrode, or to other uncontrolled factors) may have masked potential increase in spontaneous and evoked firing rates. In addition, we should keep in mind that based on cortical evoked potentials recorded in awake animals, there was no increase in evoked potentials amplitude 1 day after an acoustic trauma despite the fact that increases were detected 4 h after trauma (Sun et al., 2008).

Also, we could not detect any effect on the width of tuning curves as reported in some studies (Noreña et al., 2003). This is not totally surprising given that, as shown by Scholl and Wehr (2008), a complex disruption of the excitation/inhibition balance occurs immediately after the acoustic trauma, which selectively increases and decreases the strength of inhibition at different positions within the receptive field. In addition, besides the small size of the neuronal population we have studied, it is possible that the lack of effect on the frequency tuning stems from differences between anesthetized and unanesthetized conditions. Changes in the excitation/inhibition balance converging onto a particular cell are obviously important for controlling the shape of the frequency and intensity tuning but, in awake animals, these effects can be masked by the state of vigilance, the animals' attention and/or the concentrations of neuromodulators at the vicinity of the recorded cells (review in Edeline, 2003, 2012). Despite the fact that the EEG was collected to make sure the recording sessions took place in waking conditions, it was neither possible to evaluate the animals' attention to the different stimuli nor to evaluate its level of arousal or alertness.

Conflicting results were previously described on the latency changes observed immediately after an acoustic trauma. On the one hand, Noreña and colleagues show examples of shorter latencies and shorter response durations in the cat auditory cortex within the 2–4 h following trauma (see the example in Figure 12 in Noreña et al., 2003). On the other hand, in the rat auditory cortex, Scholl and Wehr (2008) showed that membrane potential responses were delayed and prolonged throughout the receptive field in the tens of minutes following the trauma. The methodological differences explaining this discrepancy remain unknown. However, when auditory cortex neurons were tested 2–5 months after the acoustic trauma, the mean response latency was 5 ms longer in traumatized cats than in normal cats (Eggermont and Komiya, 2000). In any case, this suggests that the time at which cortical neurons are tested after an acoustic trauma is potentially a crucial factor. This urges for additional studies during which the time course of cortical changes will be evaluated. Several mechanisms, having different time courses, can underlie our results: the effects on the response latency were transient whereas the effects on the cortical thresholds, although attenuated, were long lasting, potentially reflecting that TTS and PTS have different consequences on the responses of auditory cortex neurons.

Whether or not the cortical alterations reported here reflect residual responses or a central (thalamo-cortical) plasticity is a challenging question. When testing the different levels of the auditory system after hearing loss, evidence for responses having the properties of residual responses come from studies performed at the cochlear nucleus (Rajan and Irvine, 1998) and inferior colliculs levels (Irvine et al., 2003), whereas the map changes reported at the cortical and thalamic level were considered as real reorganizations (Robertson and Irvine, 1989; Rajan et al., 1993; Kamke et al., 2003). As shown in Figure 3, some of our recording sites recovered CFs with threshold close from the pre-trauma level which suggests that the changes described here result from plastic changes and not from residual responses.

### Responses to natural stimuli after acoustic trauma

Many previous studies have stressed that the discrimination performance of auditory cortex neurons are better when based on the temporal organization of spike trains rather than when based on the average firing rate (e.g., see Schnupp et al., 2006; Huetz et al., 2009; Gaucher et al., 2013a). At presentation of conspecific vocalizations, we found here that there was no significant change in firing rate but a decrease of the *CorrCoef* (for the cells with CF > 5 kHz), an index quantifying the spike-timing reliability (Figure 6). This means that at the level of individual cells, the trial-to-trial reliability of evoked responses is impaired after the trauma. The latency shift, and more importantly, the increase in response duration (Figure 7) observed from the onset PSTH indicate that the synchronization of cortical cells is also impaired by the trauma. Altogether, these results point out that important aspects of neuronal responses to communication sounds are modified after hearing loss. A puzzling result is the fact that the responses latency was only increased the day after trauma when tested with pure tones, whereas it was increased for several days when tested with communication sounds, suggesting that more pronounced effects can be detected with natural stimuli. Potentially, one explanation is that each pure tone activates relatively small sets of afferents converging on the recorded cell, whereas the onset of vocalizations recruits a much larger proportion of afferents converging on this cell. This is consistent with the fact that acoustic trauma induces an important loss of inhibition far away from the receptive field of the cell (Scholl and Wehr, 2008). In addition, because the response of cortical neurons to communication sounds does not depend only on the spectral content of these sounds (see Figure 1 in Gaucher et al., 2013b), one can consider that communication sounds can reveal effects that are undetectable with the test of classical tuning curves.

Often, alterations observed after hearing loss at the cortical level are interpreted as resulting from intracortical reorganizations. Based on recent results, it is unlikely that the effects described here result from an attenuation of intracortical inhibition. When reducing intracortical inhibition by pharmacological treatments, it was found that the trial-to-trial reliability and phasic inhibition were enhanced at presentation of communication sounds (Gaucher et al., 2013a). Therefore, the effects described here cannot be simply explained by a local alteration of cortical inhibition. Most likely, some of the alterations observed at the cortical level can stem from effects already occurring at sub-cortical levels. For example, after an acoustic trauma, the acute loss of the IHC ribbon synapses connected to high-threshold, low spontaneous firing rate, auditory nerve fibers may lead to decrease the synchronization of auditory nerve fiber responses. At the central level, a down-regulation of inhibition can be detected days or weeks after a noise-induced hearing loss: the level of GAD-65/67 (the enzyme responsible for the conversion of glutamate into GABA) and the levels of GABA_A_ receptor subunit α1 were found to be lowered both in inferior colliculus and auditory cortex, respectively (Wang et al., 2002; Browne et al., 2012; Kou et al., 2013).

## Conclusions

So far, very few studies have studied the consequences of hearing loss in non-anesthetized animals. Recording evoked potentials in auditory cortex 24 h after an acoustic trauma, Sun et al. (2008) did not detect increase in evoked potential amplitude despite transient increases 4 h after the trauma. The cortical recordings in performed in awake gerbils by Rosen et al. (2012) after developmental hearing loss is the only one making the link between behavior and neuronal deficits. These authors examined behavioral and neural detection thresholds for sinusoidally amplitude modulated (SAM) stimuli. In animals with bilateral conductive hearing loss, behavioral SAM detection for slow modulation (<5 Hz), but not for fast modulation (100 Hz) was impaired in hearing impaired animals. Auditory cortex neurons displayed limited impairments for static stimuli but respond poorly to slow, but not to fast, SAM tones. Comparisons between psychometric and neurometric curves (based on firing rate) indicated similar impairment at the behavioral and neural levels (Rosen et al., 2012).

To the best of our knowledge, the present study is the first one describing the consequences of partial hearing loss on the responses to communication sounds. By analyzing the responses to conspecific vocalizations we found significant alterations of parameters related with the temporal synchronization of neuronal responses. For several reasons, analyzing the cortical responses to communication sounds can be a good model for understanding how speech stimuli are processed by cortical neurons. Many studies in human now suggest that very subtle hearing loss, or sometime undetectable hearing loss, can lead to abnormal speech processing (e.g., Léger et al., 2012). This study could be a starting point to link more tightly the deficit in speech intelligibility observed in human and the alterations in cortical responses after modest hearing loss.

### Conflict of interest statement

The authors declare that the research was conducted in the absence of any commercial or financial relationships that could be construed as a potential conflict of interest.

## Footnotes

^1^Note that this value corresponds to the latency of the neuronal response as measured from the time at which the vocalizations reached a fixed threshold in terms of SPL level (the same for all vocalizations; see Methods).
